# Mentorship in Modern UK Surgical Training

**DOI:** 10.15694/mep.2017.000045

**Published:** 2017-03-08

**Authors:** Heather Stewart, Morkos Iskander

**Affiliations:** 1 St Helens and Knowsley Teaching Hospitals NHS Trust; 2 Wirral University Teaching Hospitals NHS Foundation Trust

**Keywords:** mentorship, surgery, training

## Abstract

This article was migrated. The article was marked as recommended.

Over the years, surgical training has been standardised and centralised with less emphasis on a mentorship style of training. However, having a consistent mentor during surgical training can overcome obstacles faced by trainees. It has been shown to improve career success as well as general well-being in a demanding and competitive specialty. Despite this, a mentor is not a mandatory part of surgical training with trainees struggling to identify a mentor in the present system. We suggest the introduction of a programme managed by regional deaneries which promotes the formation of a mentor and trainee relationship at the beginning of surgical training. We are confident this scheme has the potential of producing a technically able, healthy and well-rounded surgeon.

## Mentorship in Modern UK Surgical Training

Traditionally postgraduate surgical training took the form of an apprenticeship. On deciding what career path to take, surgeons would train within a specialty and form a mentor style relationship with a consultant to coach an individual through their training. Over the years, surgical training has been standardised and centralised, which has both advantages and disadvantages for trainees.

In current training, it is mandatory that trainees are allocated an Educational and Clinic Supervisor for each attachment on the rotation. The role of a supervisor is to assist in setting learning objectives and assessing an individual to ensure they obtain the required evidence to progress to the next stage of training. The role of a mentor is different; a mentor is an individual who uses their greater level of knowledge and understanding of the area they work in to support and develop a less experienced colleague. The importance of mentorship is recognised by the Royal College of Surgeons with studies demonstrating mentoring during postgraduate training has a positive impact on career success. Despite this, a mentor is not a mandatory part of surgical training and a recent survey found less than half of surgical trainees could identify a mentor.

The role of a supervisor in assessing and monitoring progression is advantageous in that it provides a structured set of objectives trainees need to obtain to progress and ensures all trainees are at the same level at each stage of training. However, this focus on assessment makes other aspects of supporting and coaching an individual more difficult. It can result in a lack of appreciation for personal strengths and weaknesses with an emphasis on standardisation rather than appreciating a trainee as an individual. This could also result in neglect of other important considerations in surgical training such as an individual’s ability to cope with the demands of training. Surgery has one of the highest drop-out rates of all specialties and studies have demonstrated high rates of burn out, anxiety and depression. The amount of surgeons seeking professional help for these issues was also found to be low. Having a mentor and forming a long-term trusting relationship could help with general well-being and help an individual to feel more confident seeking help during training. Having a mentor could also improve surgical training for those with chronic illness or disability by again providing a consistent contact for any issues during training.

Although supervisors are an integral part of training, it is not always clear how supervisors are selected or allocated and the amount of support and input supervisors provide can vary dramatically. The impact of shift-based working rotas, rather than team-based working results in a fragmented and suboptimal insight into the trainee’s progression and development may be limited. Achieving the minimum requirements by the supervisor and trainee for progression also neglects the wider development of a surgeon but is possible under current training standards. This again neglects other important aspects which make a well-rounded surgeon. A mentor could offer additional support for career progression in surgery including developing the technical and procedural skills required, audit and publications as well as personal advice on coping with the demands of surgical training.

Over recent years there has been a move to more structured assessment of competency in surgical training. Trainees now complete an on-line logbook including Work Based Assessments (WBAs). The aim of these assessments is to provide evidence of what trainees do in practice, allowing the educational supervisor to safely say the trainee has met required standards. The ability for these assessments to provide an accurate insight into a trainee’s progression has been widely debated and a recent study has suggested further work is required to investigate the role of WBAs in assessment and engagement of trainees. By having a mentor who is aware and able to comment on all aspects of a surgical trainee’s development, the assessment system has the potential to be developed and tailored to the individual and their learning needs and progress.

The role of the supervisor is often limited due to the structure of current surgical training, as trainees rotate through departments as often as every six months. Therefore, if a trainee has been able to identify and form a mentorship, either with their supervisor or a different member of the team, this unfortunately comes to an end after as little as six months. Frequent rotations can also make the completion of projects and audits difficult as the process can take considerably longer to complete. It is difficult to return to a hospital to complete an audit or project due to IT access and confidentiality concerns. Having a mentor for Core Surgical Training and then for higher surgical training, as well as supervisors during rotations may help to overcome these issues. By maintaining this relationship during training, the trainee has on-going support and guidance and would be able to maximise their potential as a surgeon.

Currently the European Working Time Directive (EWTD) is a suggested barrier to adequate surgical training. The increased number of on-call and service provision hours can prevent a trainee working regularly with a supervisor and reduce the overall number of training hours. The new Junior Doctor’s Contract could cause further fragmentation of team working as the workforce is spread to include weekends as normal working hours. Clare Marx, President of the Royal College of Surgeons, has suggested Britain leaving the European Union would allow surgeons to undergo hours of extra training without the EWTD. However, there is no evidence to suggest whether this will improve training or address further service demands required for the government’s plans for a seven day NHS. Having a mentor could lessen the effects and anxieties related to working hours and the current uncertainties regarding these demands on trainees. A possible solution could be considering the mentor and trainee relationship when developing rotas with the aim of a mentor and trainee working together regularly during elective and on-call work.

Although women now form 55% of the medical workforce, only 10.5% of consultant surgeons in England are women suggesting women still face difficulties within surgery. The presence of female role models has been shown to promote the numbers of female surgical trainees and having a mentor can help encourage women in surgical training. The Women in Surgery society appreciate the importance of role models and offer a directory of surgeons who are available for advice regarding a career in surgery for women. This could be developed further into a regional programme to allow face-to-face mentoring for female trainees.

It is self-evident a consistent mentor during surgical training can overcome obstacles faced by all trainees. It has not only been shown to improve career success but also general well-being in a demanding and competitive specialty. Despite this, a mentor is not a mandatory part of surgical training with trainees struggling to identify a mentor in the present system. We suggest the introduction of a programme managed by regional deaneries which promotes the formation of a mentor and trainee relationship at the beginning of surgical training. A local directory of suggested mentors with special interests or experiences which they are willing to share with trainees could transform the experiences of surgical trainees most in need of mentorship. The programme would offer support for mentors and a platform for continued communication if a trainee is rotating through placements or hospitals. We are confident this scheme has the potential of producing a technically able, healthy and well-rounded surgeon.

## Take Home Messages

• Mentorship in surgical training has been shown to improve career success as well as general well-being in a demanding and competitive specialty.
• Despite this, a mentor is not a mandatory part of surgical training with trainees struggling to identify a mentor in the present system.
• We suggest the introduction of a programme managed by regional deaneries which promotes the formation of a mentor and trainee relationship at the beginning of surgical training.

## Notes On Contributors

Heather Stewart

Peninsula Medical School Graduate 2013 and currently a Core Surgical Trainee in Mersey Deanery with an interest in modern surgical training and teaching.

Morkos Iskander

Current Urology trainee at ST5 level with a keen interest in medical education.
