# Supplementary material for: Mentorship in Modern UK Surgical Training
Source: MedEdPublish (2016). 2017 Mar 8;6:45. [Version 1] doi: 10.15694/mep.2017.000045 (PMC10885241; doi:10.15694/mep.2017.000045)
Supplement: Peer review history

# Reviewer report: Reviewer response for version 1

**DOI:** 10.21956/mep.19412.r28494

**Published:** 2017-03-09

**Authors:** Michael SH Wan

**Affiliations:** 1 University of Notre Dame

This review has been migrated. The reviewer awarded 4 stars out of 5 This paper model of mentorship was well described and could be applicable to other disciplines. Would be interested to look into the dynamics and interactions between the mentor and trainees; any incidences of conflicts and negative feedback. Workplace based assessment by the supervisor that could also be the mentor would also affect the relationship and the assessment outcome. A longitudinal study to look at the outcome and effect of this program would be valuable.

Reviewer Expertise:

NA

No decision status is available

# Reviewer report: Reviewer response for version 1

**DOI:** 10.21956/mep.19412.r28491

**Published:** 2017-03-08

**Authors:** John Cookson

**Affiliations:** 1 University of Worcester

This review has been migrated. The reviewer awarded 4 stars out of 5 I agree with that; it's part of a wider process that requires numbers rather than words to describe the training process (and much else besides).

Reviewer Expertise:

NA

No decision status is available

# Reviewer report: Reviewer response for version 1

**DOI:** 10.21956/mep.19412.r28492

**Published:** 2017-03-08

**Authors:** Trevor Gibbs

**Affiliations:** 1 AMEE

This review has been migrated. The reviewer awarded 4 stars out of 5 An interesting opinion piece covering an evolving issue. I think that the authors make a very good case for mentorship, not just in surgery but I feel in many training posts. Sadly and although this commentary relates to the UK situation, the situation overseas is not much better. The authors point out several reasons why mentorship has reduced in popularity and I would agree it is not just one single factor and I would agree with them that educational supervision, although very important in its own right, has taken over from mentorship.As someone who qualified in 1973 and with having a family, has frequently seen the health service from a patient perspective, it has been very sad to see the demise of the closeness that used to exist between the healthcare providers, the camararderie that existed between the various levels of those providers and the " mentorship", all-be informal that went on, not just medical but pastoral and career developmental.I am sure that there are good examples of mentoring; I would hope that the authors can follow this paper up with a more structured piece of research that now looks and critically appraises those models with the possibility of implementing and evaluating their own ideas.

Reviewer Expertise:

NA

No decision status is available

# Reviewer report: Reviewer response for version 1

**DOI:** 10.21956/mep.19412.r28493

**Published:** 2017-03-08

**Authors:** Richard Hays

**Affiliations:** 1 James Cook University

This review has been migrated. The reviewer awarded 4 stars out of 5 This paper presents interesting and useful perspectives on mentorship, with which I largely agree. However, the problems with mentorship schemes is that they seem to depend on the chemistry between individuals. Where this works it can be very beneficial. Long term relationships and support networks can be formed. The big question is: how do we make 'mandatory' mentorship work regardless of the relationship? Some of the answer may lie in better matching of people, but more research is needed into just what mentorship is and how it can be made more reliably helpful.

Reviewer Expertise:

NA

No decision status is available
